# A Nutrient-Deficient Microenvironment Facilitates Ferroptosis Resistance via the FAM60A–PPAR Axis in Pancreatic Ductal Adenocarcinoma

**DOI:** 10.34133/research.0300

**Published:** 2024-02-02

**Authors:** Hong Pan, Yue Sun, Li-Heng Qian, Ying-Na Liao, Yan-Zhi Gai, Yan-Miao Huo, Zuo-Qing Li, Hui-Zhen Nie

**Affiliations:** ^1^ State Key Laboratory of Systems Medicine for Cancer, Shanghai Cancer Institute, Ren Ji Hospital, School of Medicine, Shanghai Jiao Tong University, Shanghai 200240, China.; ^2^ Department of Biliary-Pancreatic Surgery, Ren Ji Hospital, School of Medicine, Shanghai Department of Biliary-Pancreatic Surgery, Ren Ji Hospital, School of Medicine, Shanghai Jiao Tong University, Shanghai 200127, China.; ^3^ Innomodels Biotechnology Co., Ltd., 51 Xinpei Road, Jiading District, Shanghai, China.

## Abstract

Ferroptosis, a nonapoptotic form of cell death, is an emerging potential therapeutic target for various diseases, including cancer. However, the role of ferroptosis in pancreatic cancer remains poorly understood. Pancreatic ductal adenocarcinoma (PDAC) is characterized by a poor prognosis and chemotherapy resistance, attributed to its high Kirsten rats arcomaviral oncogene homolog mutation rate and severe nutritional deficits resulting from a dense stroma. Several studies have linked rat sarcoma (RAS) mutations to ferroptosis, suggesting that inducing ferroptosis may be an effective strategy against oncogenic RAS-bearing tumors. We investigated the role of Family With Sequence Similarity 60 Member A (FAM60A) in this study, a protein closely associated with a poor prognosis and highly expressed in PDAC and tumor tissue from Kras^G12D/+^;Trp53^R172H/+^; Pdx1-Cre mice, in regulating ferroptosis, tumor growth, and gemcitabine sensitivity in vitro and in vivo. Our results demonstrate that FAM60A regulates 3 essential metabolic enzymes, ACSL1/4 and GPX4, to protect PDAC cells from ferroptosis. Furthermore, we found that YY1 transcriptionally regulates FAM60A expression by promoting its transcription, and the Hippo-YY1 pathway is restricted in the low-amino-acid milieu in the context of nutrient deprivation, leading to downstream suppression of peroxisome proliferator-activated receptor and ACSL1/4 and activation of GPX4 pathways. Importantly, FAM60A knockdown sensitized PDAC cells to gemcitabine treatment. A new understanding of FAM60A transcriptional regulation pattern in PDAC and its dual function in ferroptosis reliever and chemotherapy resistance is provided by our study. Targeting FAM60A may therefore offer a promising therapeutic approach for PDAC by simultaneously addressing 2 major features of the disease (high RAS mutation rate and tumor microenvironment nutrient deficiency) and preventing tumor cell metabolic adaptation.

## Introduction

Pancreatic ductal adenocarcinoma (PDAC) is a highly lethal malignancy with a 5-year survival rate of only 8% [1]. Despite extensive research efforts, a number of factors contribute to the poor prognosis of PDAC, including delayed diagnosis, a dearth of reliable biomarkers, and resistance to conventional treatment. The current standard of the cure for PDAC includes surgery, chemotherapy, and radiation therapy, but these approaches have had limited success in improving patient outcomes. A new therapeutic target must therefore be identified and more effective treatment strategies must be developed for PDAC [2,3]. Human PDAC is a highly nutrient-poor cancer that generally contains low amounts of glutamine, glucose, and glycolytic intermediates compared to adjacent normal pancreatic tissue [4]. In response to nutritional stress, tumor cells change their metabolic behavior and undergo metabolic remodeling to maintain their survival and development [5].

The cellular process of ferroptosis results in an excessive amount of lipid peroxidation downstream from metabolic malfunctions and is nonapoptotic [6]. This type of cell death is associated with mutant RAS in cancer cells and is characterized by iron-dependent lipid peroxidation [7,8]. Various pathways are involved in regulating these processes, including the iron-dependent lipoxygenase pathway, Glutathione Peroxidase 4-glutathione (GPX4-GSH) pathway, Acyl-CoA Synthetase Long Chain Family (ACSL)-polyunsaturated fatty acids (PUFAs), and Xc-cystine/glutamate antiporter [9,10]. In terms of clinical applications, ferroptosis-based therapies are still in the early stages of development [11]. However, preclinical studies have shown promising results in a variety of cancer types, including pancreatic cancer, lung cancer, and liver cancer [12]. One potential advantage of ferroptosis-based therapies is their ability to selectively target cancer cells while sparing normal cells. This is in contrast to traditional chemotherapy, which can have toxic effects on healthy tissues [13]. In addition, inducing ferroptosis in cancer cells can reinforce the efficacy of other cancer therapies, including chemotherapy and immunotherapy [5,14]. Overall, the development of ferroptosis-based therapies represents an exciting area of research in the field of cancer treatment.

In this study, on the basis of the expression levels of ferroptosis-related gene sets within the RNA-Seq data of PDAC patients collected from The Cancer Genome Atlas (TCGA) database, we performed single-sample gene set enrichment analysis (ssGSEA). We identified Family With Sequence Similarity 60 Member A (FAM60A, also called SINHCAF, SIN3-HDAC1 Complex Associated Factor) as a potential key gene that might regulate ferroptosis. FAM60A is a subunit of the Switch-independent 3-Histone deacetylase 1 (SIN3-HDAC1) deacetylation complex [15], and it can interact with complex-related proteins to regulate the cell proliferation. Loss of FAM60A causes loss of function of SIN3, a central component of this deacetylated transcriptional coregulatory complex [16,17], illustrating the vital contribution of FAM60A to the SIN3 complex. It has a zinc finger domain that binds to the promoter region of certain genes, which itself may function as a transcription factor [18]. The chromatin immunoprecipitation sequencing (ChIP-Seq) experiment revealed that many genes have FAM60A binding sites near the transcriptional start site, and there is a strong possibility that FAM60A acts more than a regulator of HDAC, given that histone acetylation levels at target gene promoters of FAM60A did not differ between WT and *Fam60a^–/–^* embryos [19]. Biddlestone et al. found that FAM60A silences the HIF-2α expression epigenetically through recruitment of the SIN3-HDAC1 corepressor factor, and this complex directly interacts with the SP1 to regulate HIF-2α expression. A functional relationship has been determined between FAM60A expression and angiogenesis (tube formation), viability, and proliferation in vitro [20].

Herein, we demonstrated that overexpression of FAM60A triggered by a low amino acid environment leads to the alteration of the GPX4 and peroxisome proliferator-activated receptor (PPAR)-ACSL1/4 signaling pathways, which, in turn, confers resistance to ferroptosis in PDAC. Our findings suggest that targeting FAM60A to induce ferroptosis could potentially inhibit tumor growth and enhance the efficacy of drug treatments; this provides an innovative therapeutic approach to PDAC.

## Results

### FAM60A is a candidate regulator of ferroptosis, and its expression is negatively correlated with PDAC prognosis

**Fig. 1.**
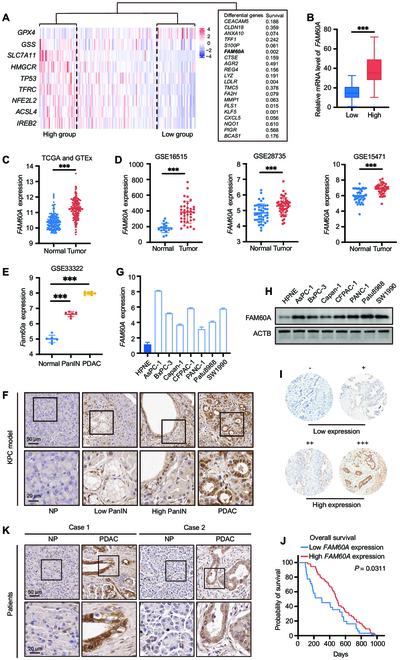
FAM60A is a candidate regulator of ferroptosis, and its expression is negatively correlated with PDAC prognosis. (A) This heatmap showed the expression differences of different gene sets between subgroups of PDAC patients identified by the ssGSEA algorithm. The 2 major subgroups were referred to as the “High-ferroptosis” and “Low-ferroptosis” groups. The top differential genes between the 2 groups and their prognosis in the TCGA database. (B) *FAM60A* expression in the “High-ferroptosis” and “Low-ferroptosis” groups. (C) *FAM60A* expression in tumor and normal pancreatic tissues in the TCGA and GTEx database. (D) *FAM60A* expression in tumor and normal pancreatic tissues in GSE16515, GSE28735, and GSE15471. (E) *Fam60a* expression in the KPC mice pancreatic tissues sequencing database (GSE33322). (F) IHC image of FAM60A expression in the KPC mice model during each pathological period. (G) qRT-PCR experiment measured *FAM60A* mRNA expression levels in the hTERT-HPNE and different PDAC cell lines; *n* = 3. (H) Western blotting experiment measured FAM60A protein expression levels in the hTERT-HPNE and different PDAC cell lines. (I) FAM60A staining was divided into 4 grades according to the depth of tissue chip IHC staining, with 2 grades with lower staining set to the low-expression group and 2 stronger grades to the high-expression group. (J) Survival analysis of *FAM60A* expression and patient survival time in TMA. (K) IHC image of FAM60A staining from patient-derived sections in PDAC and para-cancerous tissues. ***P < 0.001.

By examining the expression levels of specific genes, we identified potential subgroups based on the ssGSEA algorithm applied to the RNA-Seq data of PDAC patients from the TCGA database. We selected a set of ferroptosis-related genes and calculated the ssGSEA score for each patient using the R package gene set variation analysis (GSVA). We then performed unsupervised hierarchical clustering analysis on the ssGSEA scores, identifying 2 distinct subgroups of patients, which we refer to as the “high ferroptosis” and “low ferroptosis” groups (Fig. 1A). The top 20 genes based on fold changes were then analyzed against survival data from TCGA and 4 genes were found to be most markedly correlated with poor patient prognosis (Fig. 1A and Fig. S1). The potential role of FAM60A in ferroptosis resistance in pancreatic tumor development was ultimately investigated (Fig. 1B).

The expression profiles of *FAM60A* in PDAC and normal groups were retrieved from the Gene Expression Omnibus (GEO), TCGA, and Genotype-Tissue Expression (GTEx) databases. *FAM60A* exhibited higher expression in PDAC tissues than in normal pancreatic tissues (Fig. 1C and D). The transgenic Kras^G12D/+^;Trp53^R172H/+^; Pdx1-Cre (KPC) mouse model, which develops PDAC spontaneously, was used to examine FAM60A expression in PDAC development. A KPC mouse pancreatic tissue sequencing database (GSE33322) showed that *Fam60a* expression escalated from normal pancreatic tissue to pancreatic intraepithelial neoplasia and PDAC precursor lesions (PanIN) and PDAC (Fig. 1E), which was consistent with the immunohistochemistry (IHC) assay results (Fig. 1F). Quantitative real-time polymerase chain reaction (qRT-PCR) and Western blotting results showed that FAM60A is highly expressed in PDAC cell lines compared with the eternized human pancreatic ductal epithelial cell line hTERT-HPNE (Fig. 1G and H). Furthermore, we assessed the expression of FAM60A in a tissue microarray containing 106 samples of human PDAC tissues by immunohistochemical analysis. As determined by staining intensity and percentage of stained cells, samples were divided into 4 categories (Fig. 1I). There was a positive correlation between low *FAM60A* expression and better overall survival in survival data (Fig. 1J). IHC staining results also showed that FAM60A was up-regulated in PDAC tissues as compared with para-cancerous tissues (Fig. 1K).

Next, our study analyzed the relationship between FAM60A expression and clinical outcomes in 106 patients who had complete clinical records; the overall survival rate was inversely associated with the FAM60A expression as well as Tumor Node Metastasis (TNM) stage and vascular invasion in univariate Cox regression analysis. A multivariate Cox regression analysis identified the FAM60A expression, tumor size, lymph node metastatic disease, TNM stage, vascular invasion, and distant metastatic disease as independent predictors of overall survival for patients with PDAC (Table 1). A positive relationship between FAM60A expression and tumor size, lymph node metastasis, and TNM stage was also found (Table 2). Together, our analyses identify FAM60A as a potential ferroptosis regulator and show that its expression level is linked to clinical outcomes.

### FAM60A knockdown prevents PDAC tumor growth and promotes gemcitabine sensitivity

**Fig. 2.**
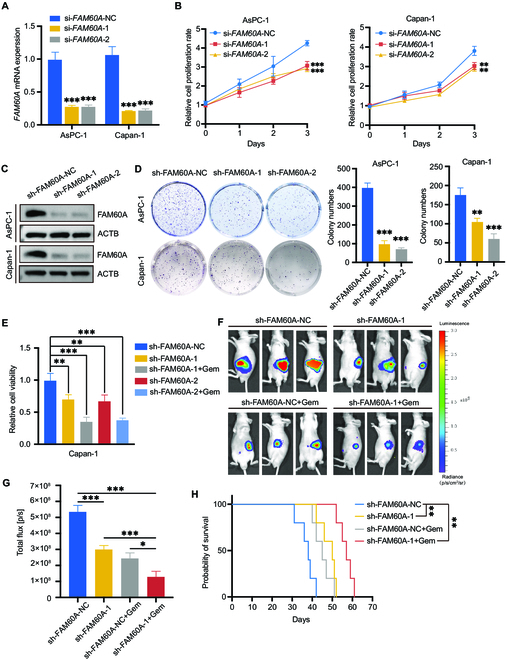
FAM60A knockdown prevents PDAC tumor growth and promotes gemcitabine sensitivity. (A) The efficiency of *FAM60A* knockdown by transfecting siRNAs was evaluated by qRT-PCR; *n* = 3. (B) CCK-8 experiments detected the efficiency of using siRNAs knockdown FAM60A on cell proliferation; *n* = 3. (C) Western blotting experiments verified the efficiency of knockdown FAM60A by shRNA. (D) Plate clonal formation experiments detected the effect of FAM60A stable knockdown on cell survival; *n* = 3. (E) Cell viability was measured in sh-FAM60A-NC, sh-FAM60A-1, and sh-FAM60A-2 cells treated with the 10 nM gemcitabine (Gem); *n* = 3. (F) Examples of bioluminescent pictures taken from luciferase-expressing Capan-1 cells that were orthotopically implanted into nude mice treated with 0.9% sodium chloride or gemcitabine (50 mg/kg). (G) Quantification of mice’s flux luminescence is conducted with IVIS. (H) Survival curves of mice implanted with sh-FAM60A-NC or sh-FAM60A-1 cells respectively treated with NaCl or gemcitabine and 0.9% NaCl (*n* = 5). ***P < 0.001, **P < 0.01, *P < 0.05.

Then, we aimed to determine if FAM60A promoted PDAC cell survival. The knockdown efficiency of *FAM60A* by siRNA was measured by qRT-PCR (Fig. 2A). Cell Counting Kit-8 (CCK-8) assays showed that cell survival was decreased in FAM60A-knockdown cells (Fig. 2B). Cell lines with stable knockdown of FAM60A were established by infection with lentivirus containing shRNA, and knockdown efficiency was validated by Western blotting (Fig. 2C). Colony formation assays showed that cell survival was descended in FAM60A-knockdown cells compared with scrambled control cells (Fig. 2D). Furthermore, knockdown of FAM60A suppressed PDAC cell resistance to gemcitabine both in vitro and in vivo (Fig. 2E to G), and the survival time of mice was correspondingly prolonged (Fig. 2H). Consequently, overexpression of FAM60A could enhance the survival of PDAC cells (Fig. S4A and B). Altogether, these results indicate that FAM60A knockdown reduced the proliferation capability of PDAC cells and restrained tumor growth, implying a tumor-promotive role of FAM60A.

### FAM60A is a repressor of ferroptosis

**Fig. 3.**
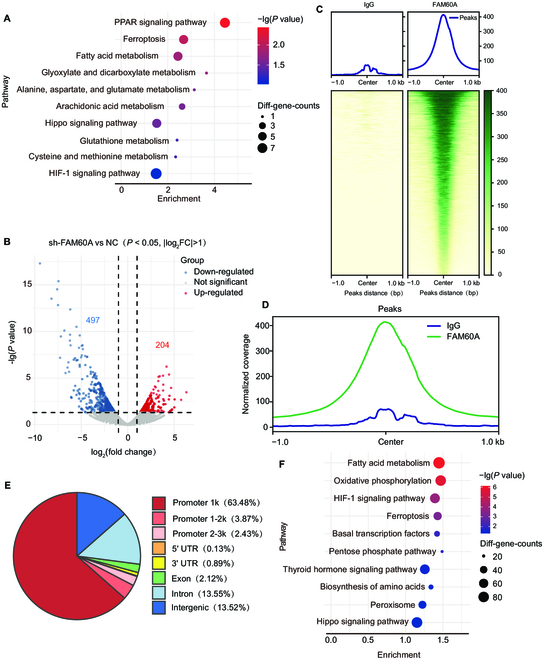
RNA-Seq and CUT&Tag data show that FAM60A regulates ferroptosis in PDAC cells. (A) KEGG pathway enrichment analysis of RNA-Seq data. (B) Differentially expressed genes up- and down-regulated in RNA-Seq data in the sh-FAM60A group and control group. (C) The heatmap showed the distribution of Reads on the genome in the CUT&Tag sequencing data. (D) Standardized reading curves of FAM60A and IgG binding peaks in CUT&Tag sequencing data. (E) The pie chart showed the genomic distribution characteristics of the FAM60A binding peak. (F) KEGG pathway enrichment analysis was performed on CUT&Tag sequencing data.

To explore the underlying pathway by which FAM60A affects PDAC progression, RNA sequencing was performed in FAM60A knockdown and scrambled control cells. On the basis of RNA-seq data, KEGG pathway enrichment analysis showed the pathways with the greatest enrichment, including the PPAR signaling pathway, ferroptosis, fatty acid metabolism, Hippo signaling pathway, glutathione metabolism, and cysteine and methionine metabolism (Fig. 3A and B). A previous study showed that, in addition to being a subunit of HDAC1, FAM60A also acts as a transcription factor [19]. PDAC cell lines were cleaved and segmented by CUT&Tag to determine genome-wide binding patterns of FAM60A. We compared all peaks in Capan-1 cell lines with those in IgG control cells (Fig. 3C and D), and most peaks were located in promoter 1k regions (Fig. 3E), suggesting that FAM60A could bind directly to the specific sequence of DNA on the target gene promoter, regulating the transcription of the downstream genes. FAM60A-bound genes were characterized through pathway enrichment analysis. Several pathways were significantly enriched in the analysis (Fig. 3F), including the fatty acid metabolism, biosynthesis of amino acids, ferroptosis, and the Hippo signaling pathway, most of which are ferroptosis-related pathways.

**Fig. 4.**
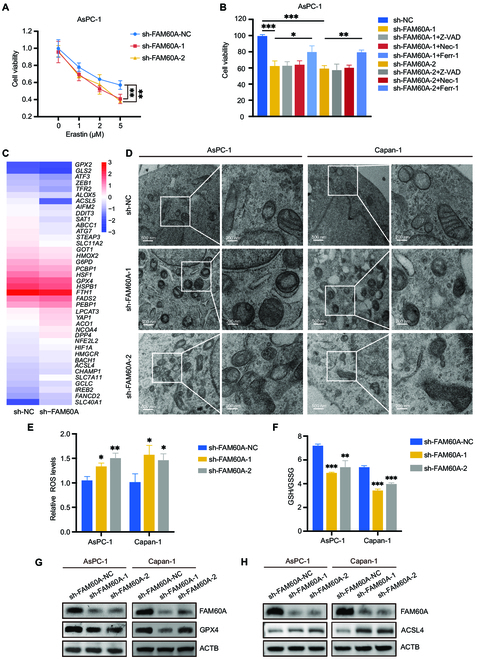
FAM60A is a repressor of ferroptosis. (A) The viability of cells treated with different concentrations of erastin in FAM60A-knockdown AsPC-1 cells was assessed; *n* = 3. (B) Effects of ferroptosis inhibitor Ferrostatin-1 (2 μM), apoptosis inhibitor Z-VAD-FMK (5 μM), and necrosis inhibitor Necrostatin-1 (2 μM) on the proliferation capacity of FAM60A knockdown AsPC-1 cells; *n* = 3. (C) Heatmap of ferroptosis-related genes changes in RNA-Seq data. (D) Transmission electron microscopy to observe the effect of FAM60A knockdown on mitochondrial morphology in PDAC cells. (E) Effect of FAM60A knockdown on ROS content in PDAC cells; *n* = 3. (F) Effect of FAM60A knockdown on GSH/GSSG content in PDAC cells; *n* = 3. (G) Western blotting experiment examined the effect of FAM60A knockdown on GPX4 protein expression levels. (H) Western blotting experiment examined the effect of FAM60A knockdown on ACSL4 protein expression levels. ***P < 0.001, **P < 0.01, *P < 0.05.

Then, we further confirmed whether FAM60A influences ferroptosis in PDAC. Functionally, a reduction in FAM60A expression significantly promoted erastin-induced cell death (Fig. 4A and Fig. S2A). Several cell death inhibitors were administered to FAM60A-knockdown cells to determine whether FAM60A impacted ferroptosis. According to our results, only ferrostatin-1 was able to prevent the decrease in cell viability caused by sh-FAM60A in Capan-1 and AsPC-1 cells; the apoptosis inhibitor Z-VAD-FMK and the necroptosis inhibitor necstatin-1s were unable to do so (Fig. 4B and Fig. S2B), illustrating that ferroptosis contributed to survival decline induced by FAM60A knockdown. Based on the RNA-Seq data, expression of ferroptosis-related gene signature was altered with FAM60A knockdown (Fig. 4C). Since damage to mitochondria is a manifestation of ferroptosis [21], FAM60A-knockdown cells had shrunken mitochondria with elevated membrane density, as revealed by transmission electron microscopy (Fig. 4D). Moreover, in FAM60A-knockdown cells, we measured GSH and reactive oxygen species (ROS) levels for lipid peroxidation to further verify the occurrence of ferroptosis. As expected, our data also showed that FAM60A knockdown markedly increased the accumulation of ROS (Fig. 4E) and decreased the content of GSH (Fig. 4F), which are both executive signals of ferroptosis [22]. GPX4 and ACSL4 have been reported to be markers of the ferroptosis pathway [23,24]. Here, we confirmed that FAM60A knockdown induced GPX4 downregulation (Fig. 4G and Fig. S2C) and ACSL4 up-regulation at both the mRNA and protein levels (Fig. 4H and Fig. S2D). Moreover, we conducted an examination of the protein expression levels of these ferroptosis-marker proteins, as well as the ROS and GSH levels, in the FAM60A-overexpressed PDAC cells (Fig. S4C to E). All the data convincingly demonstrate that FAM60A renders PDAC cells more resistant to ferroptosis.

### FAM60A constrains ferroptosis via the PPAR and GPX4 signaling pathway

**Fig. 5.**
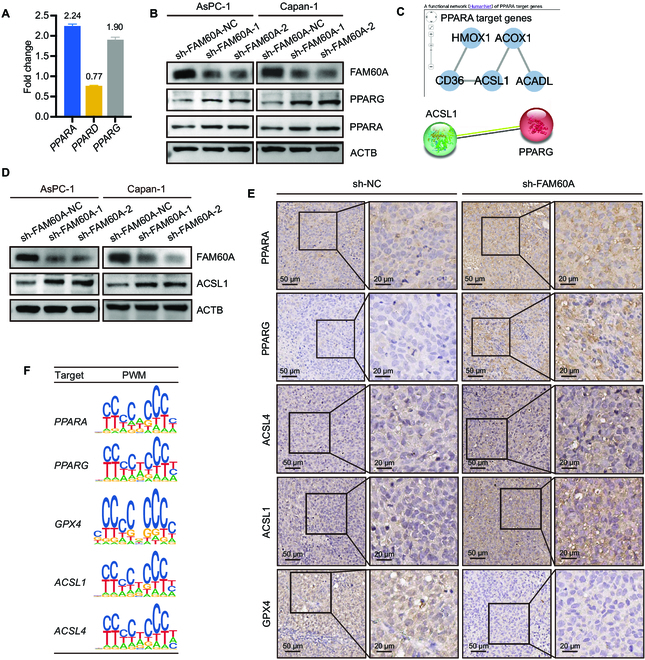
FAM60A constrains ferroptosis in PDAC cells through the PPAR and GPX4 pathway. (A) *PPARA, PPARG,* and *PPARD* fold changes in RNA-Seq data. (B) Western blotting experiment examined the effect of FAM60A knockdown on PPARA and PPARG protein expression levels. (C) The ChIP-Seq database predicted regulatory genes downstream of PPAR. (D) Western blotting experiment examined the effect of FAM60A knockdown on ACSL1 protein expression levels. (E) An IHC staining of paraffin sections from nude mouse orthotopic xenograft tumors was performed in order to determine whether FAM60A knockdown affected PPARA, PPARG, ACSL1, ACSL4, and GPX4 levels. (F) Schematic diagram of FAM60A binding site with promoter regions of PPARA, PPARG, ACSL1, ACSL4, and GPX4 as a transcription factor in CUT&Tag sequencing data.

We next sought to determine the critical point at which FAM60A is engaged in ferroptosis regulation. We found that the most enriched pathway in the RNA-Seq data was the PPAR signaling pathway, which is a nuclear hormone receptor-containing pathway that regulates lipid metabolism and plays a profound role in ferroptosis [25,26]. The PPAR family consists of PPARA, PPARD (also known as PPARB), and PPARG. Analysis of RNA-Seq data found that *PPARA* and *PPARG* were up-regulated in FAM60A knockdown group cells, while *PPARD* changes were not obvious (Fig. 5A). Western blotting experiments showed that FAM60A knockdown up-regulated PPARA and PPARG in PDAC cells (Fig. 5B).

**Fig. 6.**
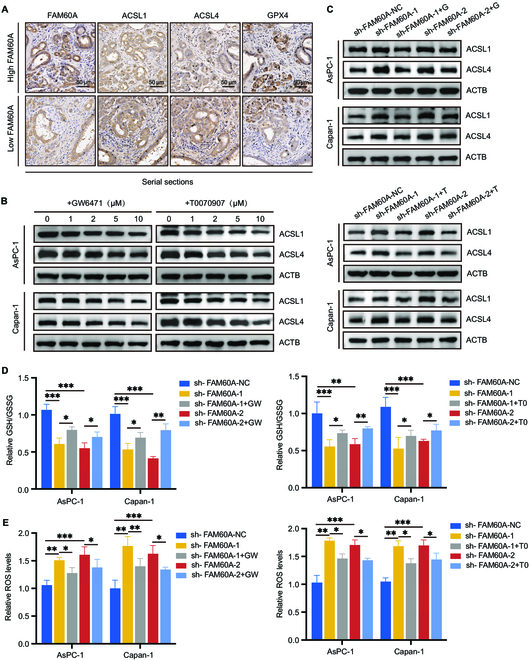
Pharmacological experiments verify that FAM60A regulates ferroptosis via the PPAR pathway. (A) A comparison of serial tissue sections of PDAC patient samples stained with IHC revealed differences in expression of ACSL1, ACSL4, and GPX4. (B) Wild-type PDAC cells were treated with 2 inhibitors, and Western blotting experiments detected changes in the expression of downstream ACSL1 and ACSL4. (C) Western blotting experiments detected expression changes of ACSL1 and ACSL4 caused by FAM60A knockdown and PPAR inhibitor treatment. (D) FAM60A-knockdown cells were treated with 2 inhibitors, and detect changes in GSH/GSSG levels in each group of cells; *n* = 3. (E) FAM60A-knockdown cells were treated with 2 inhibitors, and detect changes in ROS levels in each group of cells; *n* = 3. ***P < 0.001, **P < 0.01, *P < 0.05.

In the ChIP-Seq database (http://chip-atlas.org/; https://www.grnpedia.org/trrust/), we found that both PPARA and PPARG might transcriptionally regulate ACSL1 expression, another member of the ACSL family (Fig. 5C), which has been reported to mediate αESA-induced ferroptosis in breast cancer cells [27]. Western blotting experiments showed that FAM60A knockdown was also able to increase ACSL1 expression (Fig. 5D). IHC staining of paraffin sections of orthotopic transplantation tumors displayed deeper staining of PPARA, PPARG, ACSL4, and ACSL1 in the FAM60A-knockdown group. Conversely, GPX4 staining was lighter in the sh-FAM60A group than in the control group (Fig. 5E). In addition, we observed the FAM60A-binding peaks in the promoter regions of PPARA, PPARG, ACSL1, ACSL4, and GPX4 by analyzing CUT&Tag sequencing data (Fig. 5F), revealing a role of FAM60A in transcriptional regulation of these genes. Additionally, we stained serial tissue sections of PDAC samples from the FAM60A high-expression and low-expression groups by IHC, and observed differences in ferroptosis-related protein expression between the 2 groups. As a result, ACSL4 and ACSL1 expressions were lower in the high-FAM60A-expression group, GPX4 was higher, and the low-FAM60A-expression group tended to be opposite in nature (Fig. 6A). The outcomes were consistently replicated in consecutive serial sections of pancreatic tissue from KPC mice (Fig. S5).

Next, pharmacological treatments were used to further validate that PPAR regulates ferroptosis by ACSL4/1. PDAC cells were treated with GW6471, a specific inhibitor of PPARA, and T0070907, a specific inhibitor of PPARG, and we found that their downstream ACSL1 and ACSL4 expression levels were reduced (Fig. 6B). Adding 2 PPAR inhibitors separately to FAM60A-knockdown cells restored the overexpression of ACSL1 and ACSL4 induced by FAM60A knockdown (Fig. 6C). The addition of 2 inhibitors to sh-FAM60A cells restored the changes in GSH levels and ROS levels induced by FAM60A knockdown (Fig. 6D and E), and correspondingly, the addition of 2 inhibitors to sh-FAM60A cells also attenuated the effects of FAM60A knockdown on cell growth (Fig. S2E). The above data strongly proved that FAM60A knockdown induces ferroptosis in PDAC cells mainly through the PPAR-ACSL4/1 pathway and GPX4.

### A low-amino-acid environment activates YY1 and initiates *FAM60A* transcription

**Fig. 7.**
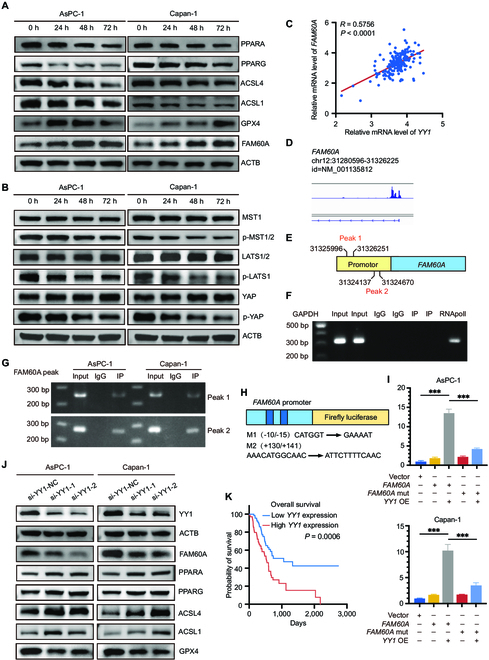
A low-amino-acid environment activates YY1 and initiates *FAM60A* transcription. (A) Western blotting assays detected changes in protein expression of FAM60A, PPARA, PPARG, ACSL1, ACSL4, and GPX4 in low-amino-acid environments. (B) The key Hippo pathway proteins were detected in low-amino-acid environments through Western blotting experiments. (C) Correlation analysis of *YY1* and *FAM60A* mRNA expression using the TCGA database. (D) The database predicted that YY1 has binding peaks in the *FAM60A* promoter region. (E) Peaks indicate where YY1 binds to the promoter of *FAM60A*. (F and G) ChIP-PCR analysis: Promoter binding interaction of YY1 with *FAM60A*. Using *FAM60A* promoter primers and negative control primers, DNA was immunoprecipitated. (H and I) Testing the ability of YY1 to bind to *FAM60A* promoter and initiate gene expression in PDAC cells using luciferase assays; *n* = 3. (J) Western blotting experiments detected protein expression level changes of FAM60A and its downstream genes by interfering with YY1 expression using siRNAs. (K) Kaplan–Meier survival curve of *YY1* in TCGA database. ***P < 0.001.

Nutrients, such as amino acids, are depleted in PDAC tumors in comparison with adjacent nonneoplastic pancreatic tissue [28], and developing strategies to cope with nutrient deprivation has led PDAC tumors to develop a variety of strategies to meet their amino acid requirements [29,30]. The Hippo pathway can be arrested and the transcription factor YY1 can be activated by low-amino-acid environments, according to our previous studies [31,32]. The expression changes in FAM60A, the downstream PPAR pathway, and ferroptosis-related genes at the mRNA and protein level were investigated at 0 h, 24 h, 48 h, and 72 h after PDAC cells were cultured in low glutamine medium to mimic a low-amino-acid environment. Under low-amino-acid conditions, PPARA, PPARG, ACSL1, and ACSL4 expression were improved, and GPX4 expression was diminished (Fig. 7A and Fig. S3A and B). Moreover, Western blotting experiments verified that the low-amino-acid environment can inhibit the Hippo signaling pathway (Fig. 7B and Fig. S3C).

The mRNA expression levels of *YY1* and *FAM60A* were analyzed in the TCGA and GEO database, and their expression exhibited a linear relationship (Fig. 7C and Fig. S6A). IHC staining results on consecutive tissue sections from pancreatic cancer patients revealed that tissues with high YY1 expression exhibited elevated expression of FAM60A, and vice versa, further underscoring the expression correlation between YY1 and FAM60A (Fig. S6B). Importantly, the ChIP-sequence database showed 2 prominent peaks in the *FAM60A* promoter regions with YY1 binding (Fig. 7D and E). The *FAM60A* promoter region primer and negative control primer were designed for the predicted binding site, and the ChIP-PCR experiment using YY1 antibody determined that YY1 was directly bound to the *FAM60A* promoter region (Fig. 7F and G); moreover, the dual-luciferase reporter gene experiments indicated that the promoter region of *FAM60A* was directly occupied by YY1 (Fig. 7H and I). Correspondingly, YY1 knockdown was able to decrease FAM60A expression (Fig. 7J). In the TCGA database, YY1 expression was correlated positively with poor prognosis in patients (Fig. 7K). Collectively, these results indicate that amino acid deficiency triggers FAM60A up-regulation via Hippo-YY1, subsequently altering PPAR-ACSL1/4 and GPX4 expression.

### Schematic diagram

**Fig. 8.**
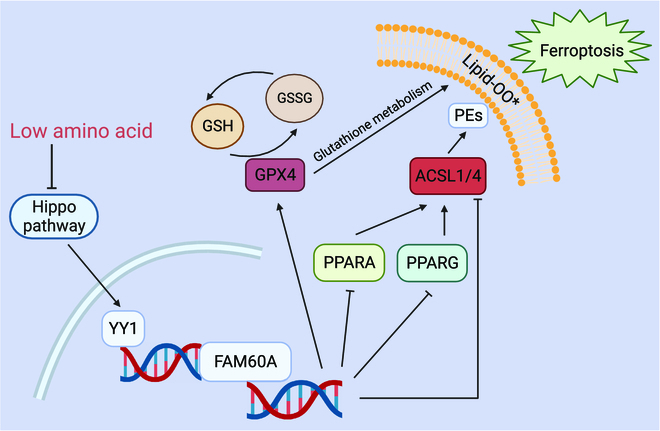
A schematic diagram illustrating the potential mechanism by which FAM60A regulates ferroptosis in PDAC cells. PE, phosphatidyl ethanolamine.

We elucidate the role of FAM60A in PDAC ferroptosis in the present study (Fig. 8). Under conditions of low amino acid availability, the Hippo pathway is suppressed and the downstream transcription factor YY1 is stimulated, which causes FAM60A expression to be up-regulated. FAM60A, functioning as a transcription factor within PDAC cells, up-regulates GPX4, a negative regulator of ferroptosis, while downregulating PPARA/G and ACSL1/4. Overall, the YY1–FAM60A axis curbs the PPAR signaling pathway, thereby inhibiting ferroptosis in PDAC cells and promoting cell survival in the context of nutrient deprivation.

## Discussion

Cancer treatment strategies typically target the selective elimination of cancer cells while minimizing harm to healthy cells. Compared to normal cells, cancer cells tend to accumulate high levels of iron [33,34]. Additionally, to sustain rapid proliferation, cancer cells require high metabolic rates, leading to elevated production of ROS [35]. As a result, tumors exhibit high levels of ROS as a distinctive characteristic. To combat this heightened oxidative stress, cancer cells must strengthen their antioxidant defense mechanisms [36]. Therapeutically targeting antioxidant defense mechanisms offers a promising approach for inducing oxidative-stress-induced cell death, including apoptosis and ferroptosis [12,37]. Moreover, ferroptosis represents a viable strategy for overcoming chemotherapy resistance [38], and combination with drugs such as temozolomide, cisplatin, haloperidol, and doxorubicin can heighten chemotherapy efficacy [39]. Despite the emergence of numerous regulatory elements associated with ferroptosis, challenges remain regarding clinical translation and application, as well as the durability of its effects [40,41]. Consequently, the identification of superior ferroptosis targets remains an essential research objective.

Engineered RAS mutant tumor cells are sensitive to ferroptosis-inducing agents like erastin and RSL3 [42]. The anticancer effects of erastin and RSL3 can be reversed by genetically or pharmacologically inhibiting RAS or downstream signaling molecules (B-Raf Serine/Threonine-Protein [BRAF], mitogen-activated protein kinase [MEK], and extracellular regulated protein kinase [ERK]). The enrichment in cellular iron pools may be due to mutant RAS signaling regulating iron metabolism genes, such as TFRC, FTH1, and FTL19 [43,44]. Lung adenocarcinoma cells harboring KRAS mutations exhibit sensitivity to ferroptosis induced by SLC7A11 inhibitors [45]. The same applies to cells derived from non-small cell lung cancer with an upstream epidermal growth factor receptor (EGFR) mutation [46]. These preclinical findings provide evidence supporting the use of ferroptosis induction as a potential treatment strategy for tumors that harbor oncogenic RAS mutations. Given the high incidence of KRAS mutations in PDAC [47,48], the identification of important ferroptosis regulators is essential for developing effective treatments [49,50]. Our study revealed that FAM60A exerts an inhibitory effect on ferroptosis in PDAC, suggesting its potential as a novel therapeutic target for this disease.

PDAC is typified by a dense stroma that results in reduced vascular supply and limited nutrients for tumor cells. In response to the nutritional deficiency of their microenvironment, tumor cells undergo metabolic reprogramming involving the activation or block of various pathways. However, the interaction between nutritional deficiency in the microenvironment and ferroptosis is not yet fully understood. Our research revealed that the Hippo pathway is repressed, leading to nuclear translocation of YY1/TAZ, which then transcriptionally up-regulates FAM60A expression in a low-amino-acid environment. FAM60A subsequently regulates the expression of PPAR-ACSL4/ACSL1, ultimately inhibiting ferroptosis. Acting as a mediator between nutritional deficiency and ferroptosis, FAM60A promotes tumor cell growth and self-protection to better adapt to adverse environments. Targeting FAM60A to induce ferroptosis may undermine the robust self-protection of tumor cells and enhance the effects of chemotherapy agents, leading to tremendous hit to tumor cell growth. We identified FAM60A as a potential therapeutic target and highlighted its association with high KRAS mutation rates and nutritional deficiencies resulting from raised matrix density, both hallmarks of PDAC. The innovative strategy of targeting FAM60A may induce more specific therapeutic outcomes than previous approaches.

Previous studies on FAM60A were mainly conducted in mice, and its role in human cells is not well understood [20]. To better understand FAM60A’s function as a transcription factor and its downstream gene targets, we performed CUT&Tag analysis in human cell lines for the first time. Our findings revealed that FAM60A is a key regulator of ferroptosis, acting as an upstream factor that modulates multiple ferroptotic pathways. Ferroptosis is driven by lipid metabolism mechanisms [51,52], and peroxidation of PUFAs, such as arachidonic acid and adrenergic acid, results in lipid bilayer destruction and impaired membrane functionality [53]. The acyl-CoA synthase ACSL4 is required to biosynthesize PUFAs in cell membranes and remodel them, while ACSL1 promotes iron uptake through its interaction with conjugated linolenic acid [27]. GPX4 is another key protein involved in ferroptosis. It is an enzyme that depends on selenocysteine and glutathione and catalyzes the conversion of lipid hydroperoxides to lipid alcohols [54]. The 2 most significant ferroptotic pathways involve ACSLs and GPX4, and this unique regulatory mechanism involving dual pathways and 3 key proteins enables FAM60A to modulate ferroptosis significantly. Targeting FAM60A for deletion is expected to induce significant ferroptosis in tumor cells, leading to substantial inhibition of tumor growth. Further studies are needed to validate the clinical relevance of our findings and to develop FAM60A-targeted therapies for PDAC.

The Hippo signaling pathway comprises a conserved set of kinases that inhibit cell growth, playing pivotal roles in cancer development, tissue regeneration, and the regulation of stem cell function. Notably, Yes-associated protein (YAP), an effector of the Hippo pathway, regulates ferroptosis [55,56]. High levels of YAP expression in most solid tumors contribute to chemotherapy resistance, making YAP a promising target for ferroptosis modulation. Intriguingly, YAP not only colocalizes with YY1 but also recruits YY1 to interact with a diverse network of genes involved in various physiological processes [31]. Additionally, YY1 has a dual role in ferroptosis regulation, inhibiting ferroptosis by suppressing sirtuins and modulating ferroptosis through autophagy control [57,58].

Despite the availability of various cancer treatment methods, the development of treatment resistance remains a significant challenge. Ferroptosis, as a therapeutic approach, holds promise in overcoming this resistance, especially given the central roles of YAP and YY1 in therapy resistance and ferroptosis modulation [59]. The complexity of YAP/YY1 signaling and ferroptosis arises from cell-type specificity and diverse regulatory mechanisms [60]. Targeted therapies against YAP/YY1 show potential in improving clinical outcomes for cancer patients. Our study reveals that the Hippo-YAP/YY1 pathway is inhibited in response to energy stress, leading to increased expression of the downstream target gene FAM60A. This, in turn, activates the key ferroptosis pathway, impacting the resistance of pancreatic cancer cells to ferroptosis. Our findings provide evidence of the Hippo pathway’s regulatory role in ferroptosis through downstream signaling.

In summary, this study highlights the crucial role of FAM60A in regulating ferroptosis, tumor growth, and gemcitabine sensitivity in PDAC. The findings suggest that targeting FAM60A could be a promising therapeutic strategy for PDAC, especially for those with oncogenic RAS mutations and severe nutritional deficits. The study also provides insights into the transcriptional regulation of FAM60A in PDAC and the underlying mechanisms by which the Hippo-YY1 pathway and downstream PPAR-ACSL1/4 and GPX4 pathways are involved in the regulation of FAM60A expression. Overall, this study sheds light on the potential of targeting ferroptosis as a therapeutic approach for PDAC and provides a new target for drug development.

## Materials and Methods

### Clinical samples

The Ren Ji Hospital harvested human tissue microarrays, PDAC tissues, and NT tissues from surgical resections of patients. Written informed consent was obtained from our patients to acknowledge the use of their resected tissues for research purposes and the study was approved by the Research Ethics Committee of Ren Ji Hospital, School of Medicine, Shanghai Jiao Tong University.

### Cell proliferation and colony formation assay

The steps in this laboratory are described in a previous article.

### Western blotting

Cells were lysed with RIPA (R0020, Solarbio, Beijing) containing Protease and Phosphatase Inhibitor Cocktail (P002, NCM Biotech, Suzhou) and collected for centrifugation. The steps in this laboratory are described in the previous article about Western experiments. Antibodies used are as follows: FAM60A (1:1,000, Novus, H00058516-B02P), GPX4 (1:1,000, Proteintech, 67763), ACSL1 (1:1,000, Proteintech, 13989), ACSL4 (1:1,000, Proteintech, 22401), PPARA (1:1,000, Proteintech, 15540), PPARG (1:1,000, Proteintech, 16643), YY1 (1:1,000, CST, #46395), MST1 (1:1,000, CST, #14946), p-MST1/2 (1:800, Abcam, ab79199), YAP (1:1,000, CST, #14074), p-YAP (1:700, CST, #13008), p-LATS1 (1:1,000, CST, #9157), LATS1/2 (1:1,000, Bethyl Laboratories, Montgomery, TX), and ACTB (1:1,000, Abways, AB0035).

### Measurement of GSH and GSSG

According to the manufacturer’s instructions, we measured total glutathione (GSH) and oxidized glutathione (GSSG) using a GSH and GSSG Assay Kit (S0053, Beyotime).

### PDAC transgenic model

The Pdx1-Cre, LSL-Trp53^R172H/+^ mice (LSL: Lox/Stop/Lox) and the LSL-Kras^G12D/+^ mice were obtained as described in the previous article in this laboratory [61].

### In vivo nude mouse orthotopic xenograft model

The steps in this laboratory are described in a previous article.

### Animal model studies

East China Normal University’s Institutional Animal Care and Use Committee approved the experiment on animals. According to the National Academy of Sciences and NIH’s *Guide for the Care and Use of Laboratory Animals* (Bethesda, MD), mice were handled and housed in accordance with the National Academy’s guidelines.

### Transfection and RNA interference

Depletion of gene expression was performed by transfecting cells with small interfering RNA (siRNA) oligonucleotides at 60% to 70% confluence. Silencing efficiency was determined by WB or qRT-PCR 72 h after transfection.

### RNA-Seq and CUT&Tag sequencing

Briefly, total RNA was extracted, purified, and quantified. Following this, the libraries were constructed according to the manufacturer’s instructions using TruSeq Stranded mRNA LT Sample Prep Kit (Illumina, San Diego, CA, USA). CUT&Tag assay was performed as described previously with modifications [62]. As recommended by Agilent, libraries were mixed and size distributions were determined using Agilent’s TapeStation 4200 analysis to achieve the desired final concentration. Following the manufacturer’s instructions, 150-bp paired-end sequencing was performed on the Illumina Novaseq 6000. CUT&Tag assay and data analysis were conducted by Jiayin Biotechnology Ltd. (Shanghai, China). Raw data have been deposited in the Sequence Read Archive database (http://www.ncbi.nlm.nih.gov/sra) (Access ID: PRJNA972122).

### Histology and immunohistochemistry

The steps in this laboratory are described in a previous article.

### Transmission electron microscopy

A 6-well plate of cell cultures was fixed for 24 h with a solution (P1126, Solarbio, Beijing) containing 2.5% glutaraldehyde in 0.1 mM phosphate buffer saline (PBS). The cells were washed in 0.1 M PBS, fixed with 1% buffered osmium, and stained with 1% Millipore-filtered uranyl acetate. The samples were incubated at 60 °C for 48 h following dehydration and embedding. Transmission electron microscope images were taken finally.

### Real-time quantitative PCR

The RNA extraction, reverse transcription, and real-time quantitative PCR steps in this laboratory are described in a previous article.

### ChIP-PCR assay

Thermo’s Pierce Agarose ChIP Kit was used for ChIP assays. DNA was immunoprecipitated from lysates after the cells were cross-linked and sonicated, and quantified by Premix Taq PCR using anti-YY1 or isotype-matched control IgG (Cell Signaling Technology).

### Dual-luciferase reporter assay

Dual-luciferase reporter assays were performed using plasmids as follows: PGL3B-vector, PGL3B-FAM60A (containing the FAM60A promoter region), and PGL3B-mFAM60A (containing the FAM60A mutant promoter region). Overexpressed-YY1 AsPC-1 and Capan-1 cells were transfected with PGL3B-promoter vector and *Renilla* luciferase expression plasmid. Dual-Luciferase Reporter Assay System (Promega) was used to analyze the cells after 24 h.

### Statistical analysis

Data were presented as the means ± SD. The SPSS software program, version 17.0 (IBM, Armonk, NY), was used for statistical analysis. Graphical representations were performed with GraphPad Prism 9 (La Jolla, CA) software. Cumulative survival curves were evaluated using the Kaplan–Meier method and tested by the log-rank (Mantel-Cox) test. The correlation between FAM60A and neighbor genes was determined using Spearman’s test. The Student’s *t* test or one-way analysis of variance was used for comparison between groups. Values of *P* < 0.05 were considered statistically significant (not significant, *P* > 0.05; *P ≤ 0.05; **P ≤ 0.01; ***P ≤ 0.001).

## Data Availability

The authors confirm that the data supporting the findings of this study are available within the article and/or its supplementary materials.
