# Supplementary material for: A Nutrient-Deficient Microenvironment Facilitates Ferroptosis Resistance via the FAM60A–PPAR Axis in Pancreatic Ductal Adenocarcinoma
Source: Research (Wash D C). 2024 Feb 2;7:0300. doi: 10.34133/research.0300 (PMC10836236; doi:10.34133/research.0300)
Supplement: Supplementary 1 — Figs. S1 to S6 Tables S1 to S4 [file research.0300.f1.zip › RESEARCH-D-23-00735-Supplementary Info.pdf]

Supplementary figure

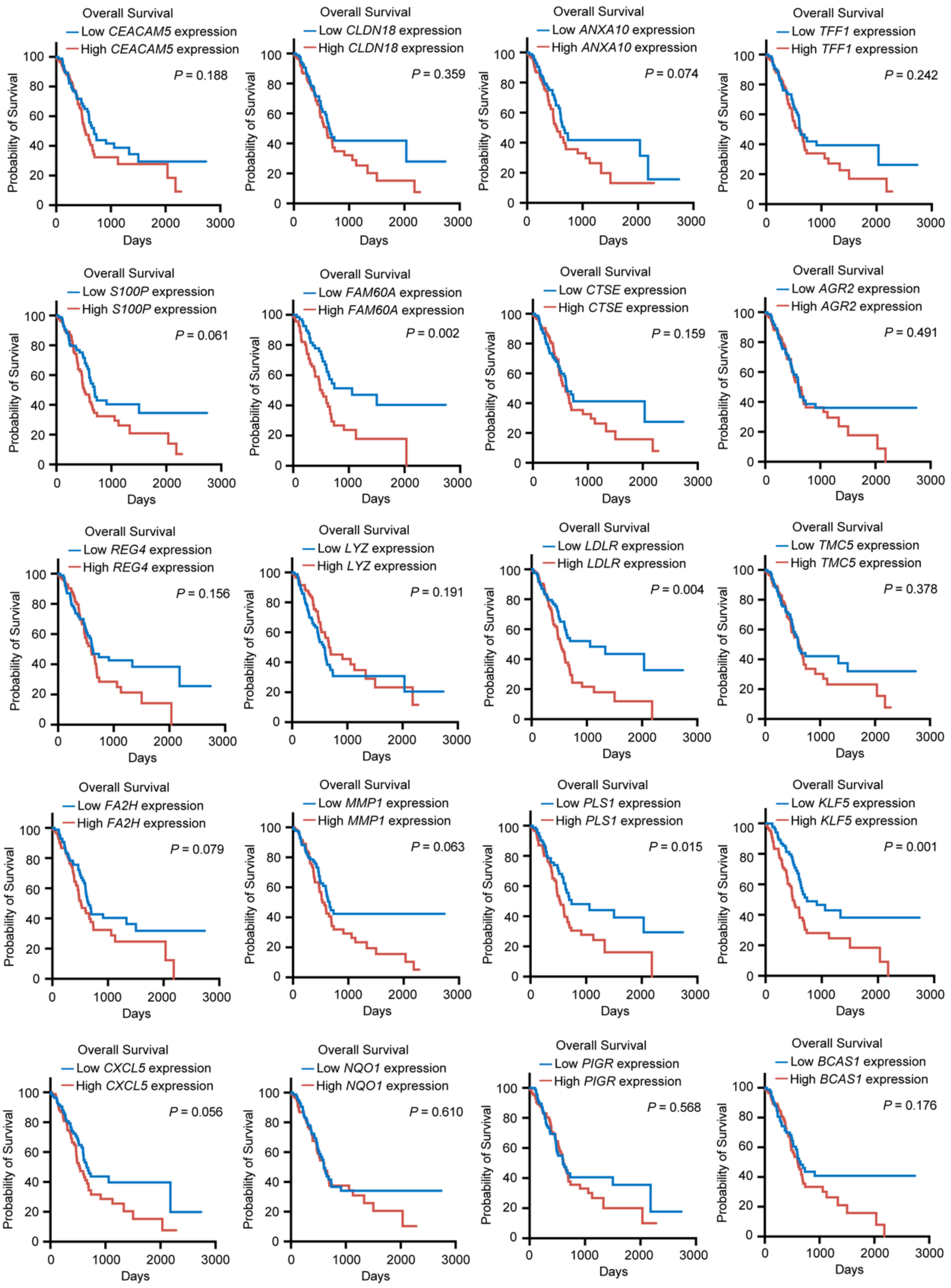

Fig.S1 Kaplan–Meier survival curve of differential genes in TCGA database.

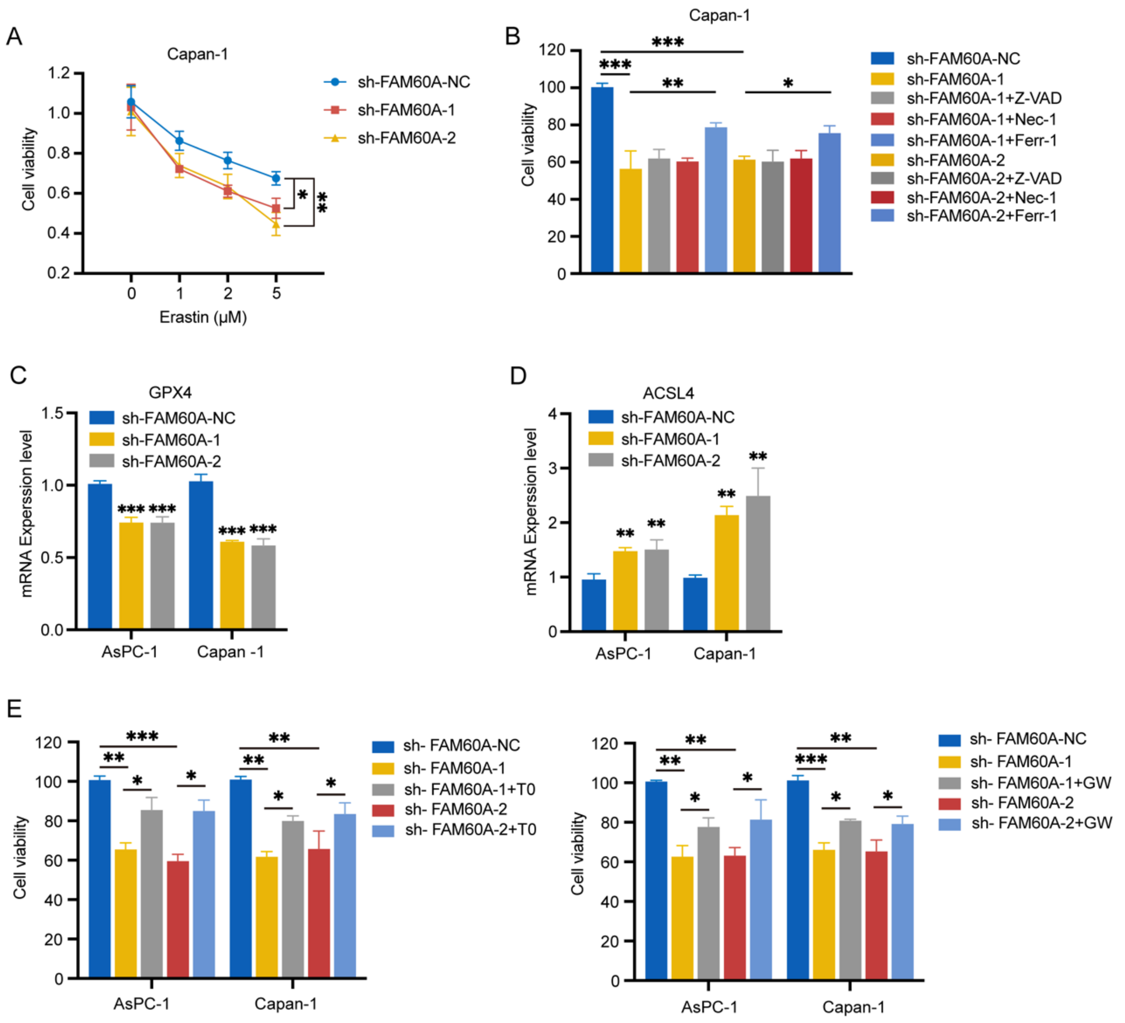

Fig.S2 A. Cell viability was assessed after treatment with different concentrations of Erastin in FAM60A-knockdown Capan-1 cells; $n = 3$. B. Effects of ferroptosis inhibitor Ferrostatin-1 (2 μM), apoptosis inhibitor Z-VAD-FMK (5 μM), and necrosis inhibitor Necrostatin-1 (2 μM) on the proliferation capacity of FAM60A knockdown Capan-1 cells; $n = 3$. C, D qRT-PCR detected the effect of FAM60A knockdown on *ACSL4* and *GPX4* mRNA levels; $n = 3$. E. FAM60A knockdown cells were treated with two inhibitors, and the CCK8 experiment examined the effect of inhibitor treatment on cell growth capacity; $n = 3$. *** $P < 0.001$, ** $P < 0.01$, * $P < 0.05$.

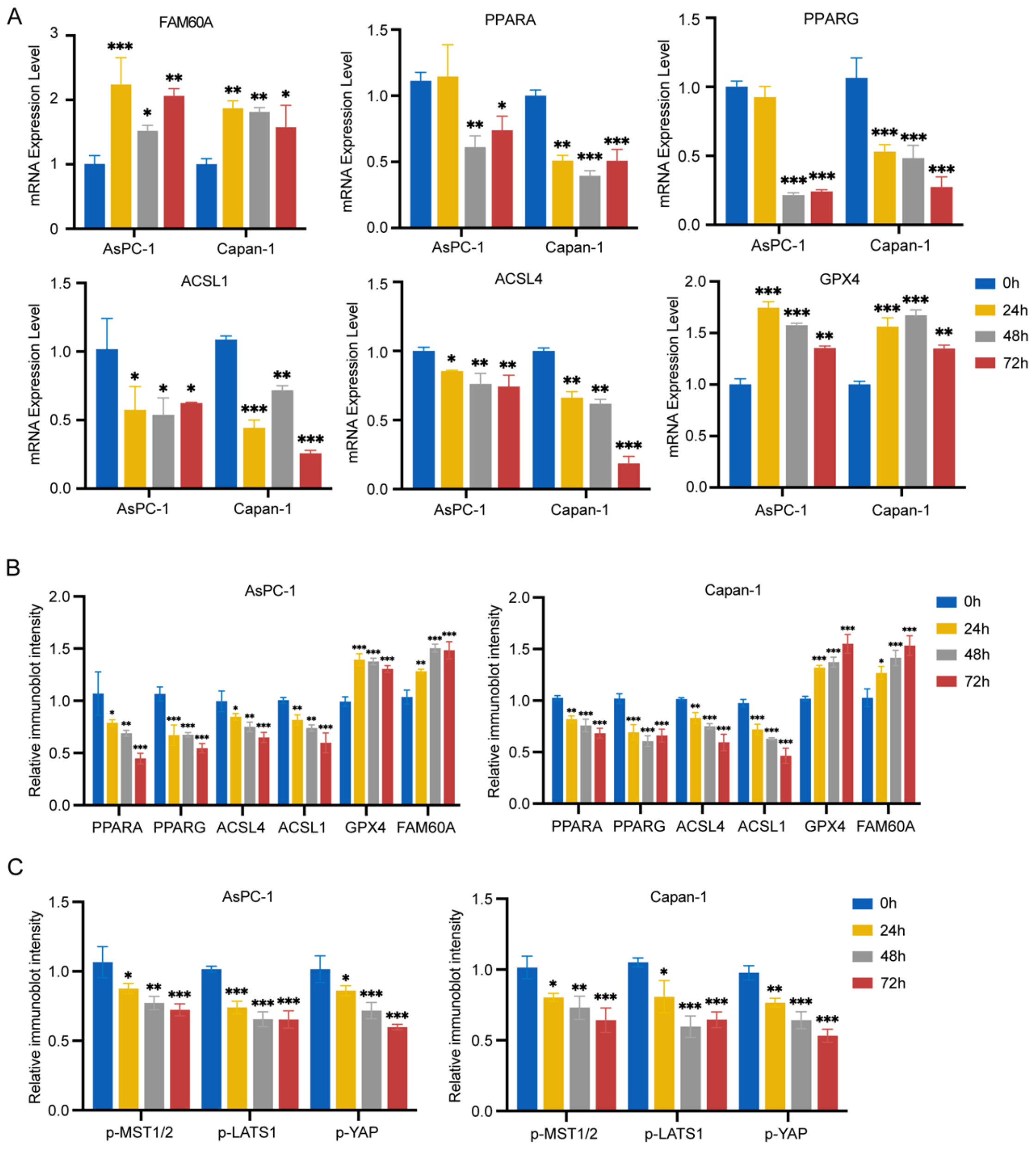

Fig.S3 A. qRT-PCR detected changes in mRNA expression of *FAM60A, PPARA, PPARG, ACSL1, ACSL4*, and *GPX4* in low-amino acid environments; $n = 3$. B. Immunoblotting quantification of PPRA, PPARG, ACSL4, ACSL1, GPX4, and FAM60A intensity; $n = 3$. C. Immunoblotting quantification of p-MST1/2, p-LATS1, and p-YAP intensity; $n = 3$. *** P ＜ 0.001, ** P ＜ 0.01, * P ＜ 0.05.

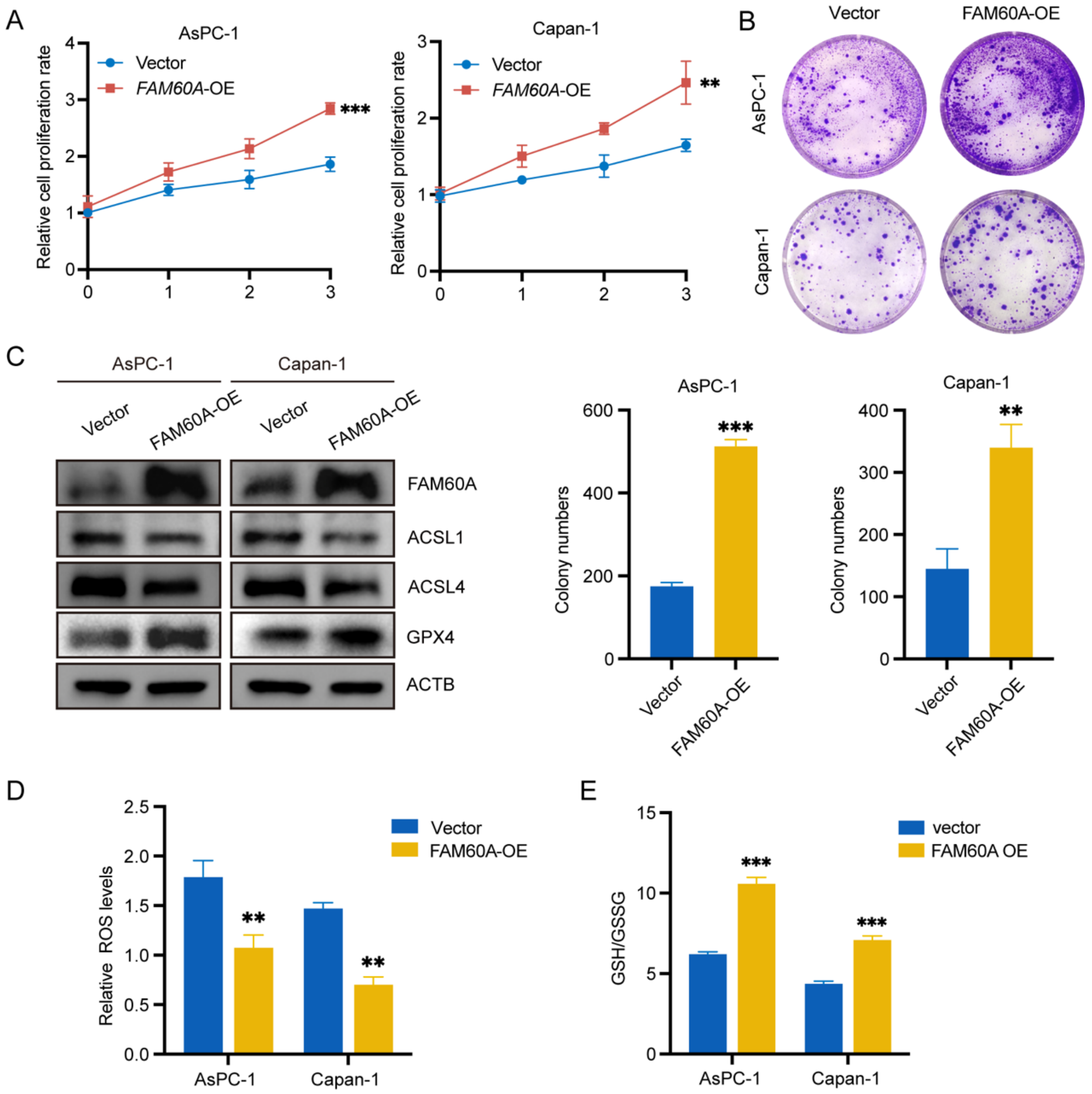

Fig.S4 A. CCK-8 experiments detected the efficiency of FAM60A overexpression on cell proliferation; $n = 3$. B. Plate clonal formation experiments detected FAM60A overexpression on cell survival; $n = 3$. C. Western blotting experiments verified the effect of FAM60A overexpression on ACSL1、ACSL4 and GPX4 protein expression levels. D/E. Effect of FAM60A overexpression on ROS and GSH/GSSG content in PDAC cells. *** $P < 0.001$, ** $P < 0.01$.

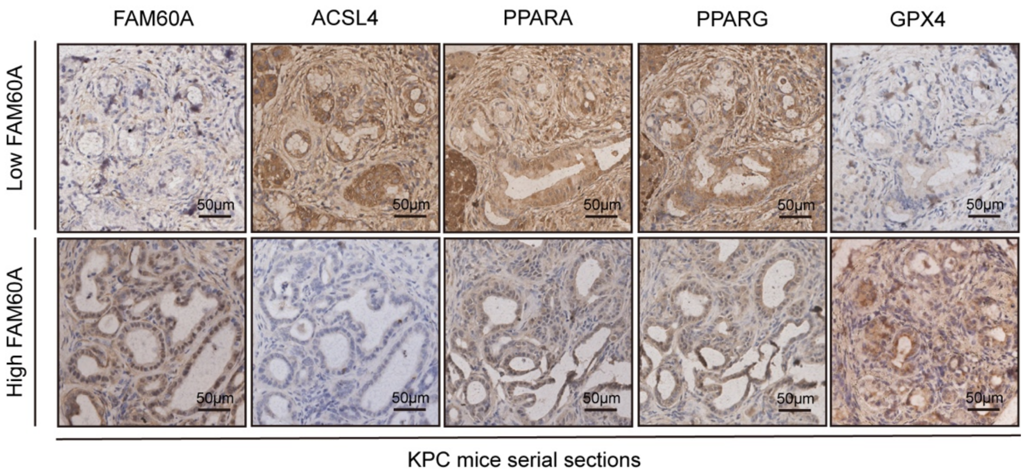

Fig.S5 A comparison of serial tissue sections of KPC mice pancreas stained with IHC revealed differences in expression of FAM60A, ACSL4, PPARA, PPARG and GPX4.

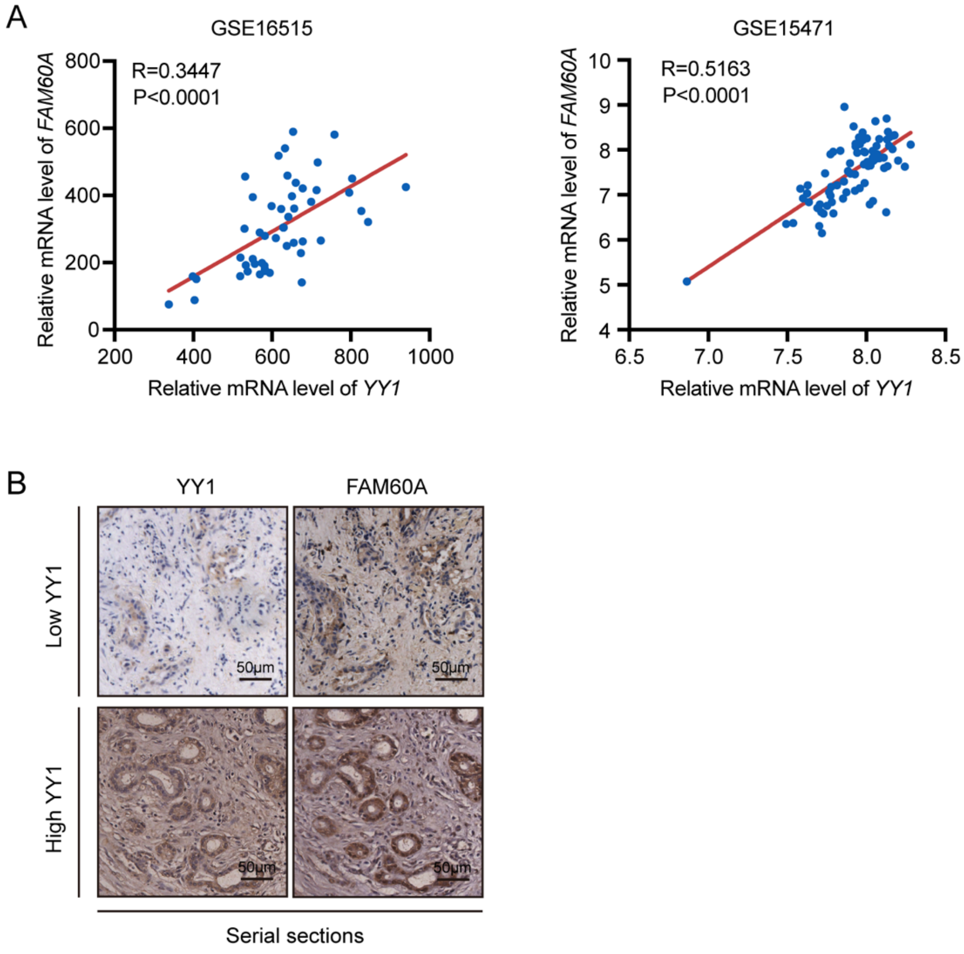

Fig.S6 A. Correlation analysis of *YY1* and *FAM60A* mRNA expression using GEO (GSE16515, GSE15471) database. B. A comparison of serial tissue sections of PDAC patient samples stained with IHC revealed differences in expression of YY1 and FAM60A.

Supplementary Table 1. Primers used for qRT-PCR in this article

Gene symbol		Sequence
18s	F	5’-TGCGAGTACTCAACACCAACA-3’
	R	5’-GCATATCTTCGGCCCACA-3’
FAM60A	F	5’-CTCCAGTTCTCGATTCACTGAC-3’
	R	5’-CGAGTCTCATGCAATCCAAAACA-3’
GPX4	F	5’-GAGGCAAGACCGAAGTAAACTAC-3’
	R	5’-CCGAACTGGTTACACGGGAA-3’
ACSL1	F	5’-CCATGAGCTGTTCCGGTATTT-3’
	R	5’-CCGAAGCCCATAAGCGTGTT-3’
ACSL4	F	5’-CATCCCTGGAGCAGATACTCT-3’
	R	5’-TCACTTAGGATTTCCCTGGTCC -3’
YY1	F	5’-ACGGCTTCGAGGATCAGATTC-3’
	R	5’-TGACCAGCGTTTGTTCAATGT-3’
PPARA	F	5’-ATGGTGGACACGGAAAGCC-3’
	R	5’-CGATGGATTGCGAAATCTCTTGG-3’
PPARG	F	5’-GGGATCAGCTCCGTGGATCT-3’
	R	5’-TGCACTTTGGTACTCTTGAAGTT-3’

Supplementary Table 2. The siRNA sequences used in this article

ID		Sequence
si-NC	F	5’-UUCUCCGAACGUGUCACGUTT-3’
	R	5’-ACGUGACACGUUCGGAGAATT-3’
si-*FAM60A*-1	F	5’-CCAAAGAUGUACCGAAGUATT-3’
	R	5’-UACUUCGGUACAUCUUUGGTT-3’
si-*FAM60A*-2	F	5’-GCUUCUGGUUCUAACAGAATT-3’
	R	5’-UUCUGUUAGAACCAGAAGCTT-3’
si-*YY1*-1	F	5'-GAAGAUGAUGCUCCAAGAATT-3'
	R	5’-UUCUUGGAGCAUCAUCUUCTT-3'
si-*YY1*-2	F	5’-UCAGUCAACUAACCUGAAATT-3'
	R	5’-UUUCAGGUUAGUUGACUGATT-3'

Supplementary Table 3. The shRNA target sequences in this article

ID	Sequence
sh-*FAM60A*-1	5’-CCAAAGATGTACCGAAGTATA-3'
sh-*FAM60A*-2	5’-GCAGAAGGAATTTAAACGTCA-3'

Supplementary Table 4. Primers used for ChIP-PCR in this article

ID		Sequence
Primer1	F	5’-CTAGAAGCATGGTGAGAGTCACTCA-3’
	R	5’-GCAAATAAGTTCGATTCAAGAGAAT-3’
Primer2	F	5’-AAGGTGTAGATTCCGCTTTCCCTCT-3’
	R	5’-GGTCTGTGGGTTCGCCTTTGAAGT-3’
Negative control	F	5’-AAGCACTTAATATGGAAAGGAAA-3’
	R	5’-TGAAGACTGAATACTGACGGGAC-3’
