# Supplementary figures and images for: A Nutrient-Deficient Microenvironment Facilitates Ferroptosis Resistance via the FAM60A–PPAR Axis in Pancreatic Ductal Adenocarcinoma
Source: Research (Wash D C). 2024 Feb 2;7:0300. doi: 10.34133/research.0300 (PMC10836236; doi:10.34133/research.0300)

Fig.1 H

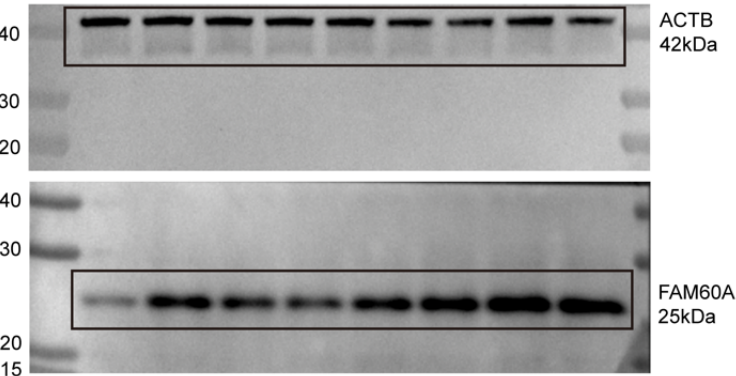

Fig.2 C

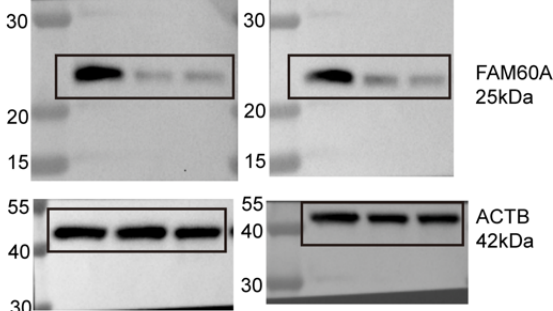

Fig.4 G

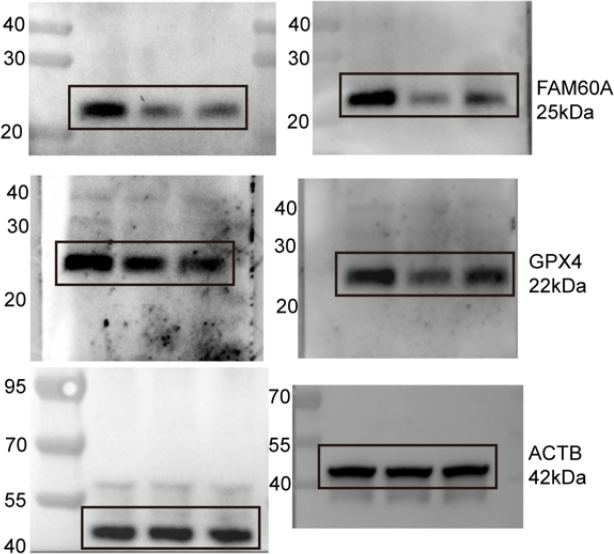

Fig.4 H

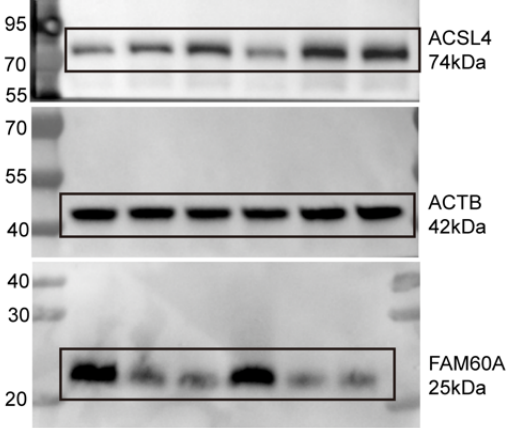

Fig.5 B

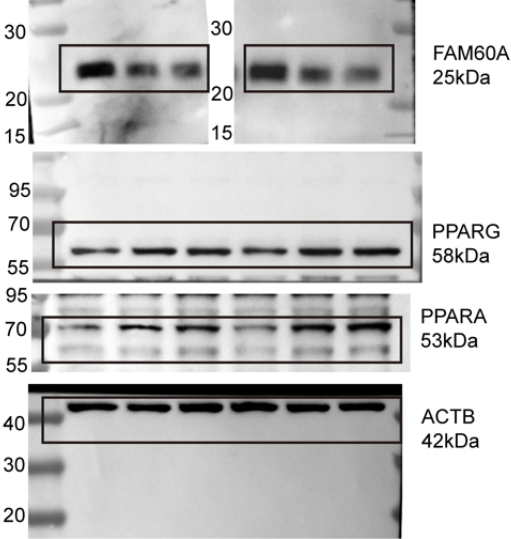

Fig.5 D

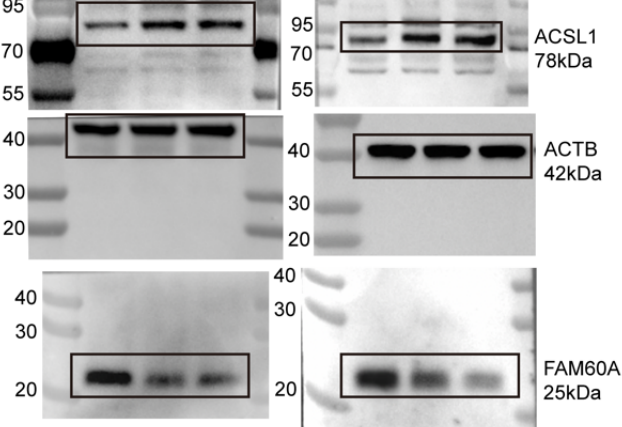

Fig.6 B

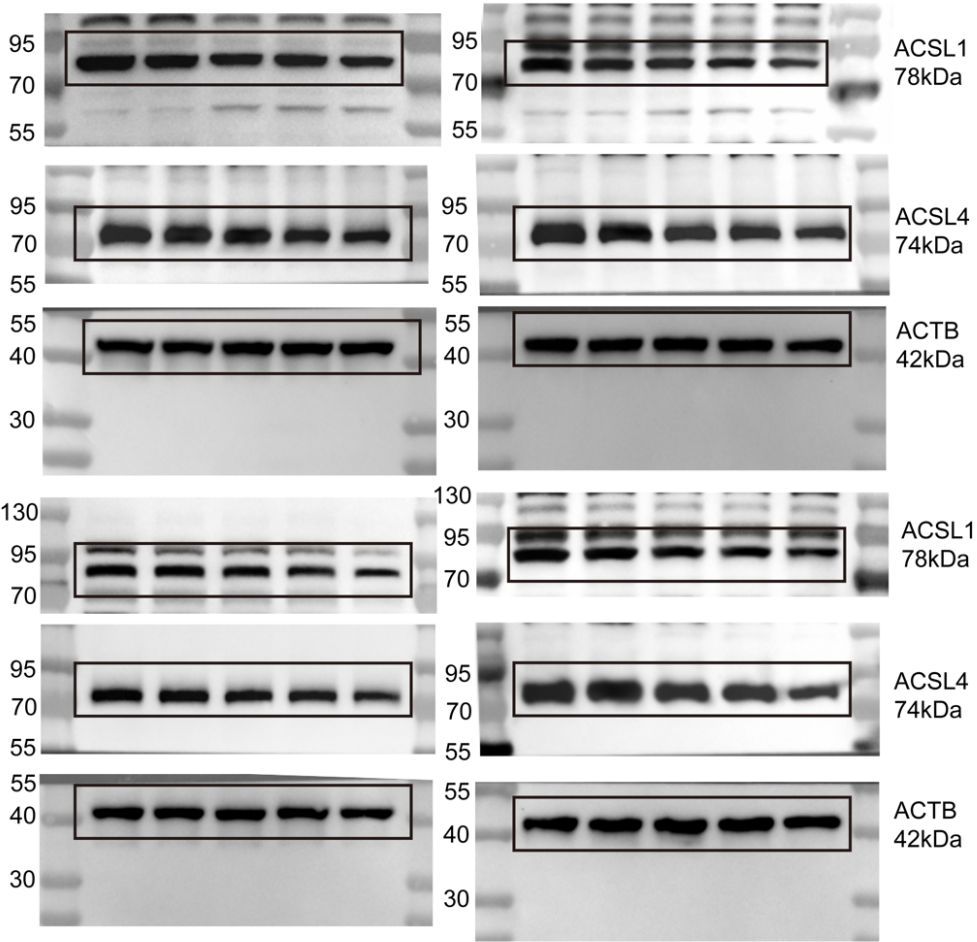

Fig.6 C

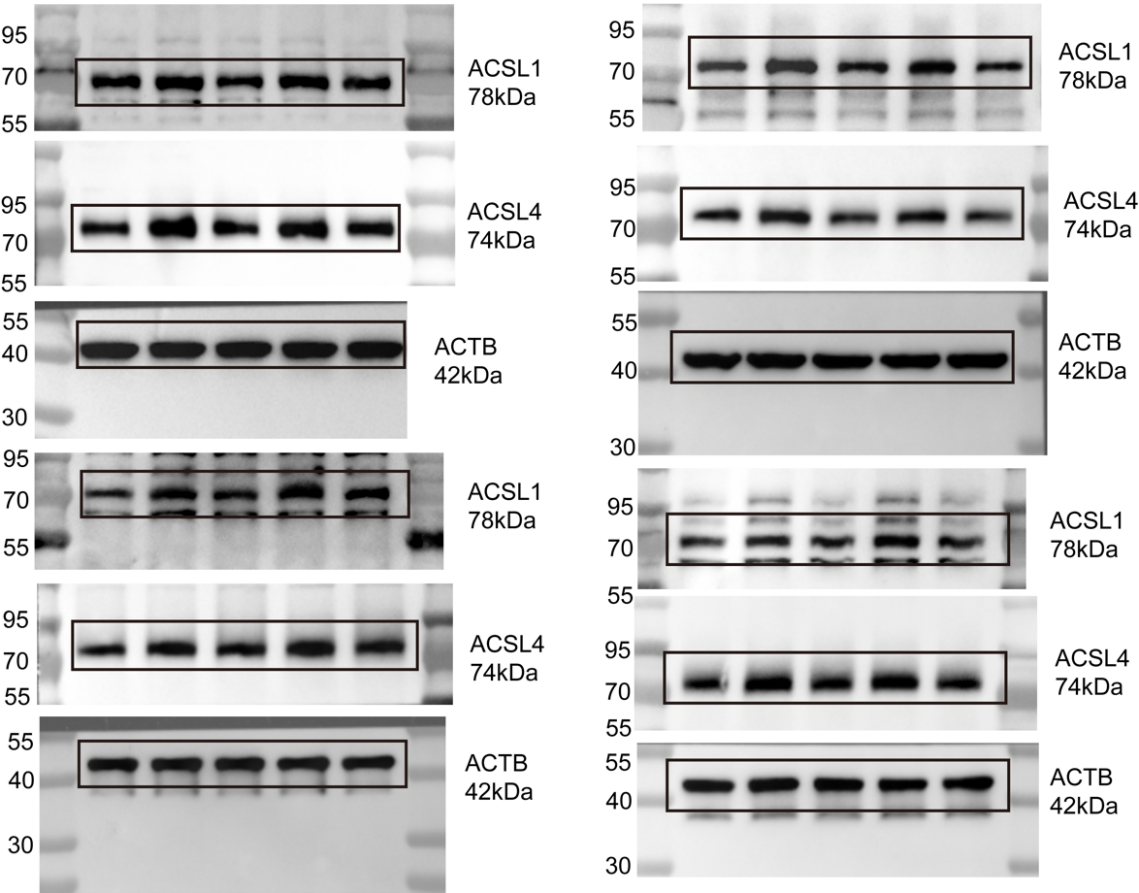

Fig.7 A

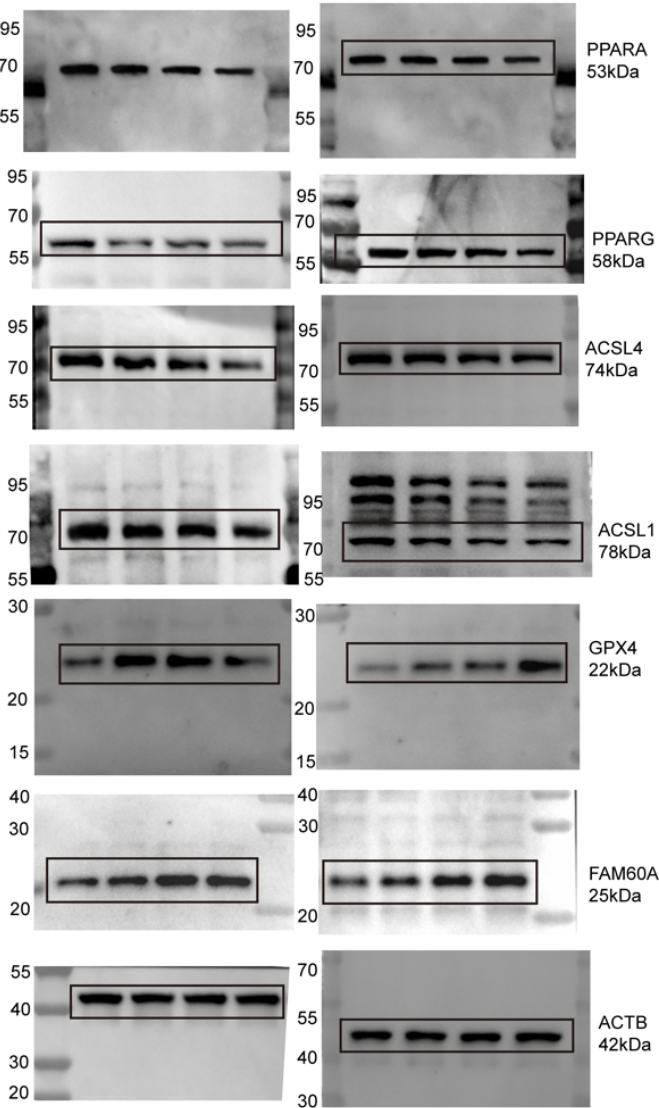

Fig.7 B

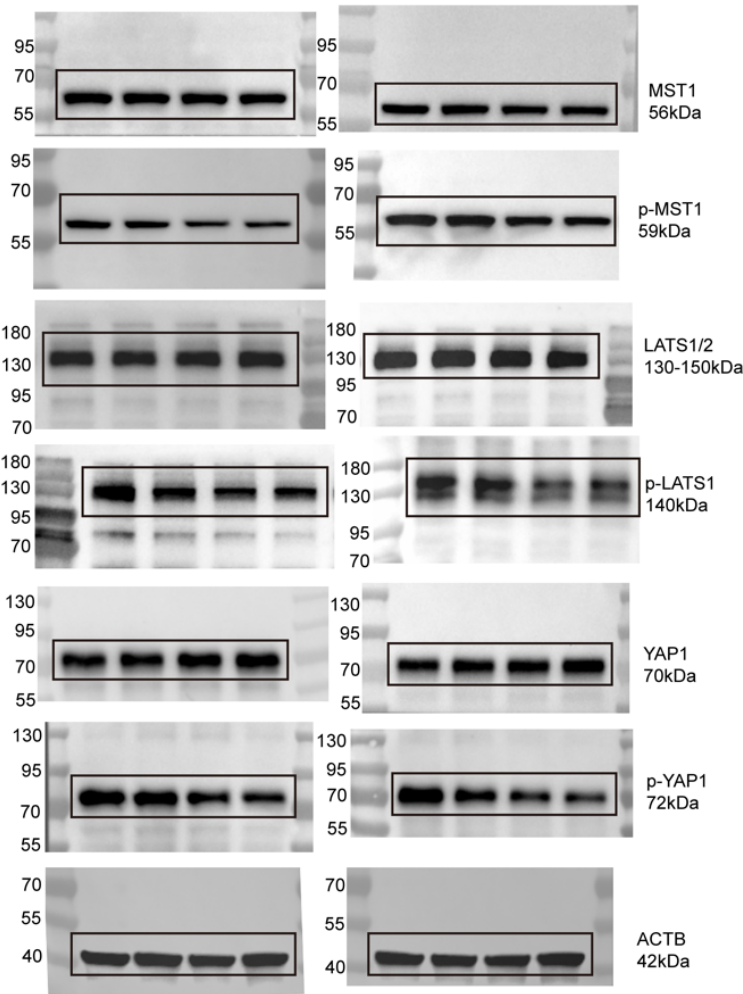

Fig.7 J

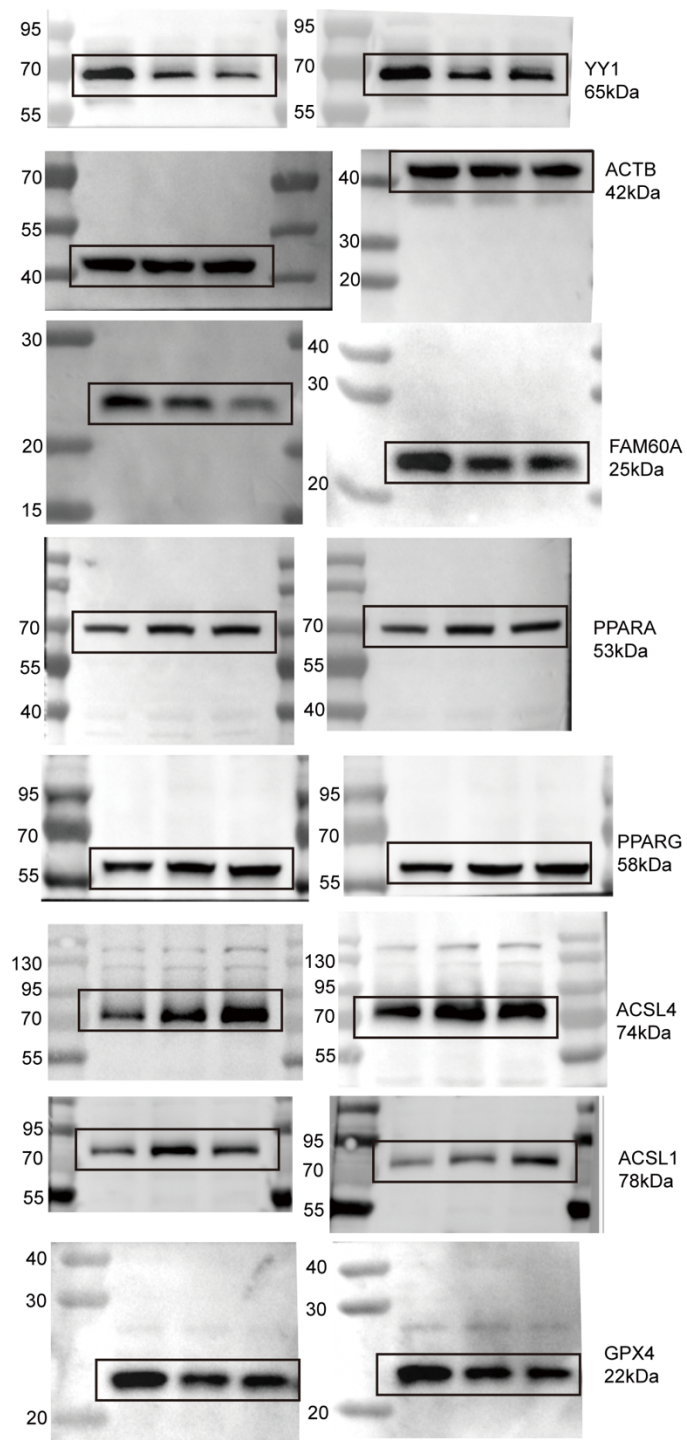

Fig.S4 C

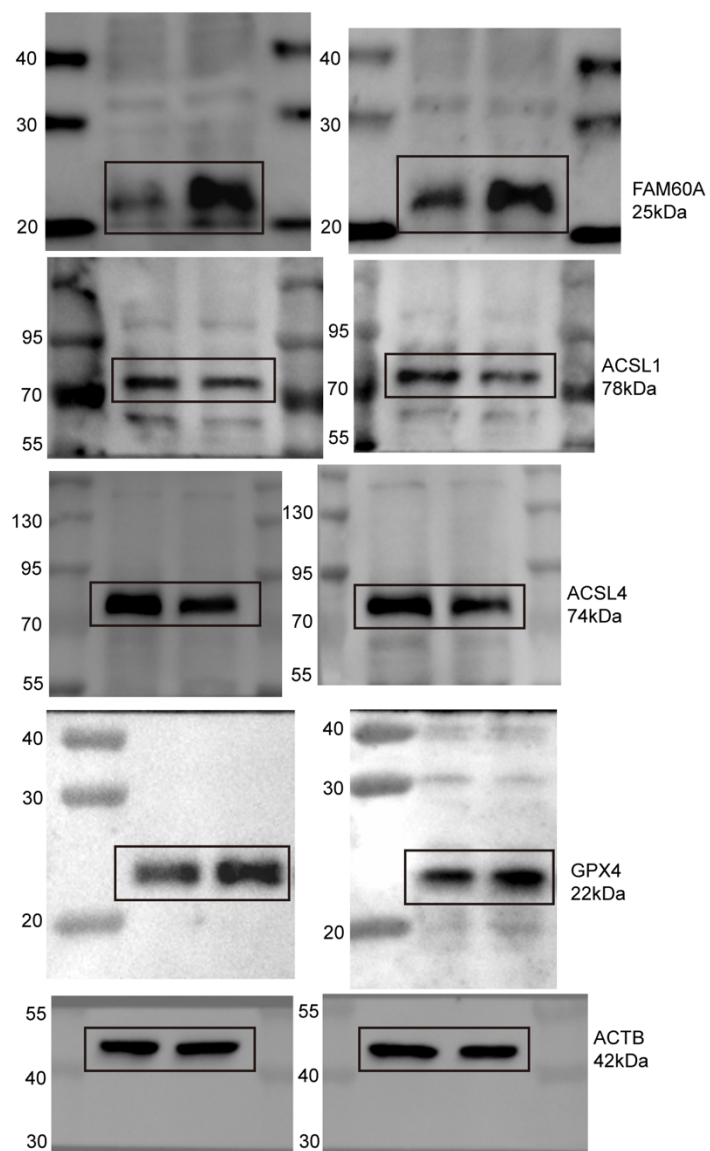

Supplement: Supplementary 1 — Figs. S1 to S6 Tables S1 to S4 [file research.0300.f1.zip › western original pic.pdf]
